# Right Atrial Angiosarcoma Presenting With Cardiac Tamponade, Hemorrhagic Pericardial Effusion, and Pulmonary Embolism: A Case Report

**DOI:** 10.7759/cureus.100780

**Published:** 2026-01-04

**Authors:** Hamoud Y Obied, Abdullah Alshutry, Ihsan Almushantaf, Galal Bashanfer, Hasan Y Guzailan, Ahmed Abdelsamae

**Affiliations:** 1 Surgery, Najran University, Najran, SAU; 2 Cardiac Surgery, King Khalid Hospital, Najran, SAU; 3 Pathology and Laboratory Medicine, King Khalid Hospital, Najran, SAU; 4 Radiology, King Khalid Hospital, Najran, SAU

**Keywords:** cardiac tamponade, malignant cardiac tumor, pericardial effusion, pericardial window, pulmonary embolism (pe), right atrial tumour

## Abstract

Primary cardiac angiosarcoma is a rare and aggressive malignancy most commonly arising from the right atrium. We report a 43-year-old male patient who had no known comorbidities and was not receiving any regular medications. He presented with abdominal and epigastric pain and was found to have severe pericardial effusion, cardiac tamponade, a large right atrial mass, hemorrhagic pericardial effusion, and bilateral pulmonary emboli. He underwent urgent pericardiocentesis followed by open-heart surgery for excision of the right atrial mass, pericardial window creation, and mediastinal lymph node biopsy. Histopathology confirmed angiosarcoma infiltrating the right atrium, pericardium, and associated thrombus. Postoperative recovery was uneventful, and oncology recommended systemic chemotherapy. This case highlights atypical presentations of cardiac angiosarcoma and outlines the surgical and diagnostic approach.

## Introduction

Primary cardiac tumors are rare, and angiosarcoma is the most common malignant subtype [1]. Histopathologic classification of these tumors follows the World Health Organization system for soft tissue tumors [2]. Systemic therapy for angiosarcoma commonly includes doxorubicin- or paclitaxel-based regimens [3], and histologic confirmation is based on characteristic malignant endothelial proliferation [4].

These tumors typically arise from the right atrial free wall and frequently present late with non-specific symptoms, pericardial effusion, or pulmonary embolism [5]. Early diagnosis is challenging due to variable presentations and the aggressive biological behavior of the disease [6]. Surgical management has been shown to provide symptomatic relief and temporary disease control in selected patients [7]. Cross-sectional imaging with computed tomography (CT) and magnetic resonance (MR) is essential for defining tumor extent and guiding operative planning [8-10]. Because of the rarity and aggressive nature of primary cardiac angiosarcoma, reporting unusual clinical presentations is important to improve understanding of diagnostic approaches and management strategies.

The primary aim of this report is to describe a rare presentation of right atrial angiosarcoma manifesting as cardiac tamponade, hemorrhagic pericardial effusion, and pulmonary embolism. The secondary aim is to highlight the diagnostic workup, surgical intervention, and clinical management that led to patient stabilization, thereby contributing to clinical knowledge and educational value for physicians encountering similar presentations [5-9].

## Case presentation

A 43-year-old male patient with no significant past medical history presented to the emergency department with a three-day history of abdominal and epigastric pain. He was alert and oriented but initially hypotensive, although his vital signs stabilized with observation. Physical examination revealed equal bilateral air entry, normal heart sounds without murmurs, a soft, non-tender abdomen, and no neurological deficits. The non-specific presentation raised concern for an underlying cardiac cause, consistent with the often vague initial symptoms reported in cardiac angiosarcoma [5,7,9].

**Figure 1:**
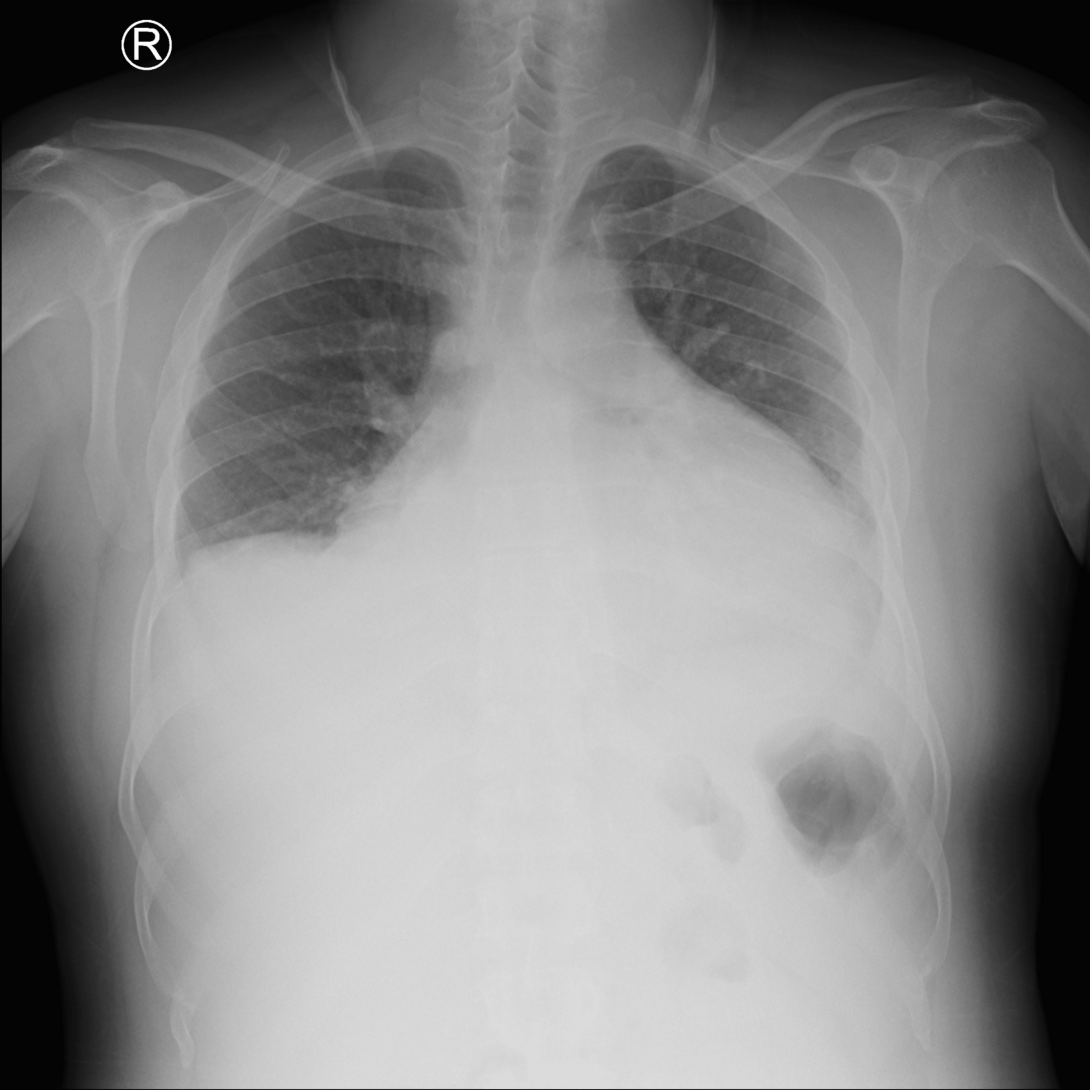
Chest X-ray showing cardiomegaly

Initial investigations showed sinus tachycardia with low-voltage complexes on electrocardiogram and a normal troponin level (0.026 ng/L). A chest X-ray demonstrated cardiomegaly, prompting further evaluation (Figure 1).

**Figure 2:**
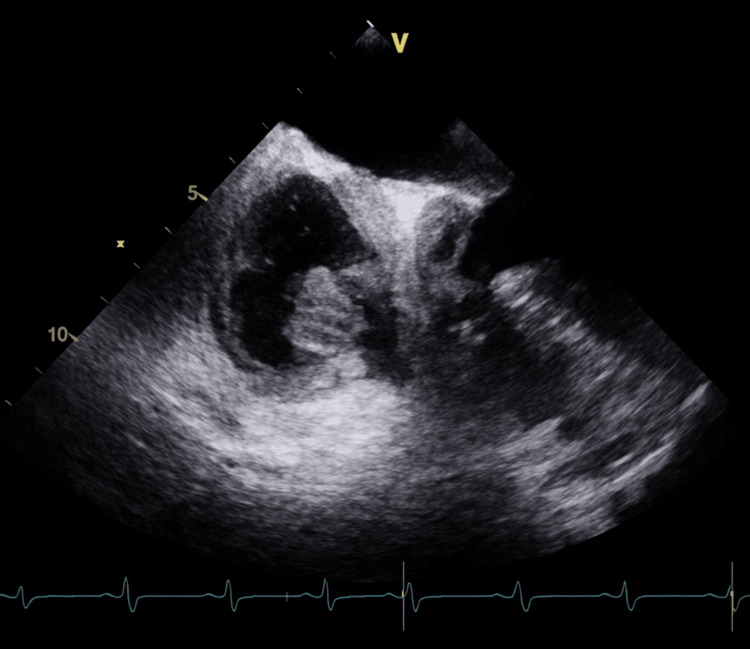
Echocardiography showing right atrial mass

**Figure 3:**
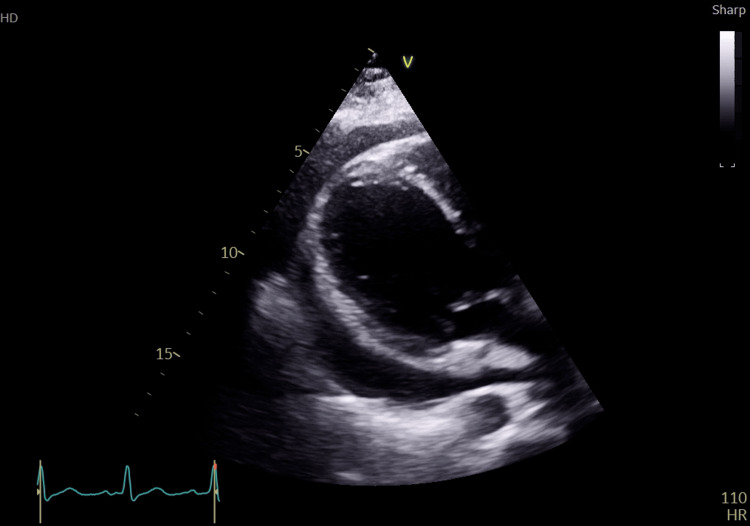
Echocardiography showing pericardial effusion

Transthoracic echocardiography revealed a large infiltrative right atrial mass measuring approximately 16 cm, attached to the lateral free wall, with severe pericardial effusion causing tamponade physiology. Mild tricuspid regurgitation, pulmonary artery systolic pressure of 30-40 mmHg, and small mobile masses in the pulmonary artery were also noted (Figures 2, 3) [5].

On August 6, 2025, the patient underwent urgent pericardiocentesis, which drained hemorrhagic exudative fluid. Cytology demonstrated atypical cell clusters with rare rosette-like structures, and immunohistochemistry was positive for pan-cytokeratin and calretinin but negative for chromogranin, leukocyte common antigen (LCA), and carcinoembryonic antigen (CEA), raising strong suspicion for malignancy [4].

**Figure 4:**
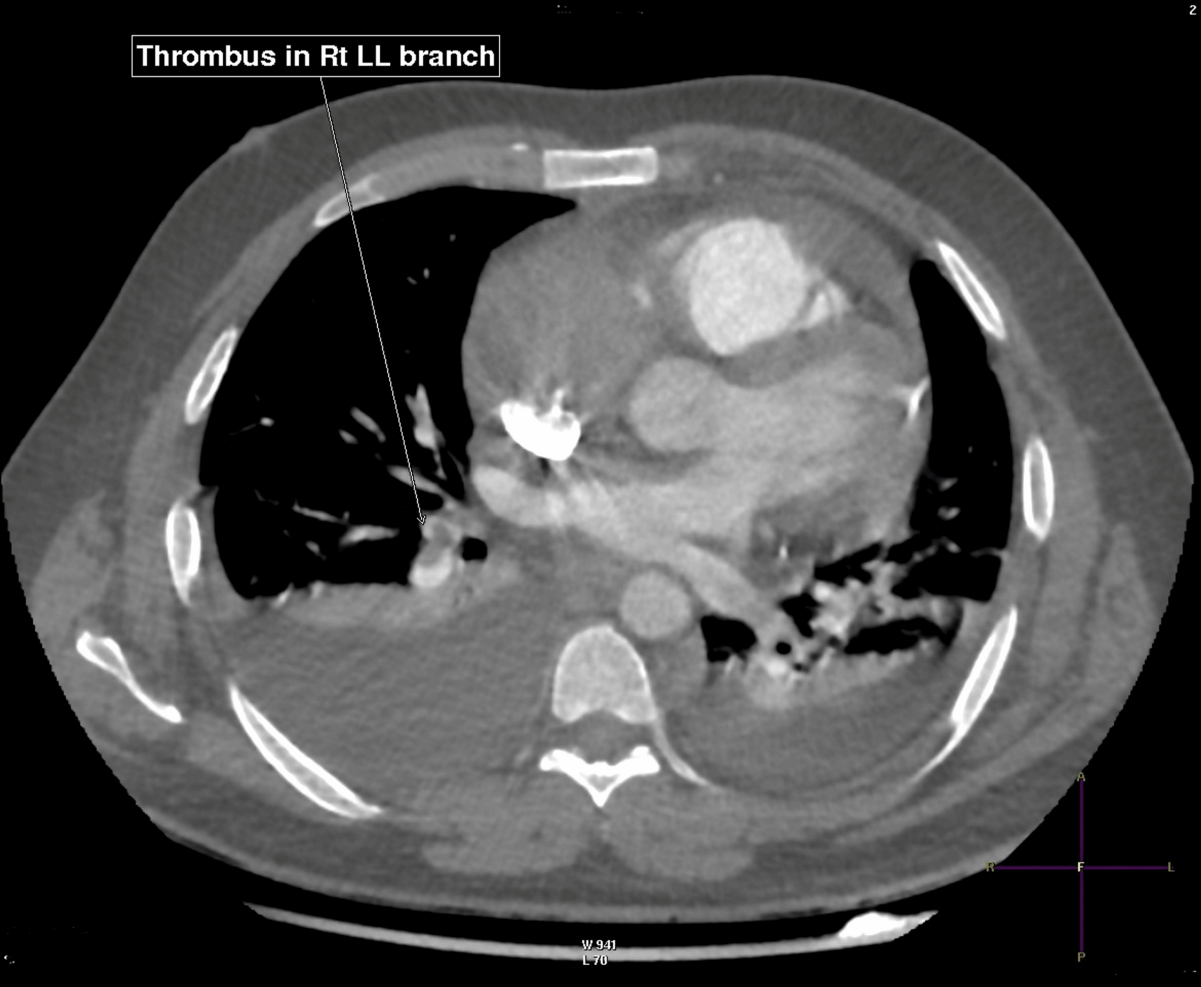
Computed tomography (CT) angiography showing thrombus in the right lower lobe (Rt LL) branch

**Figure 5:**
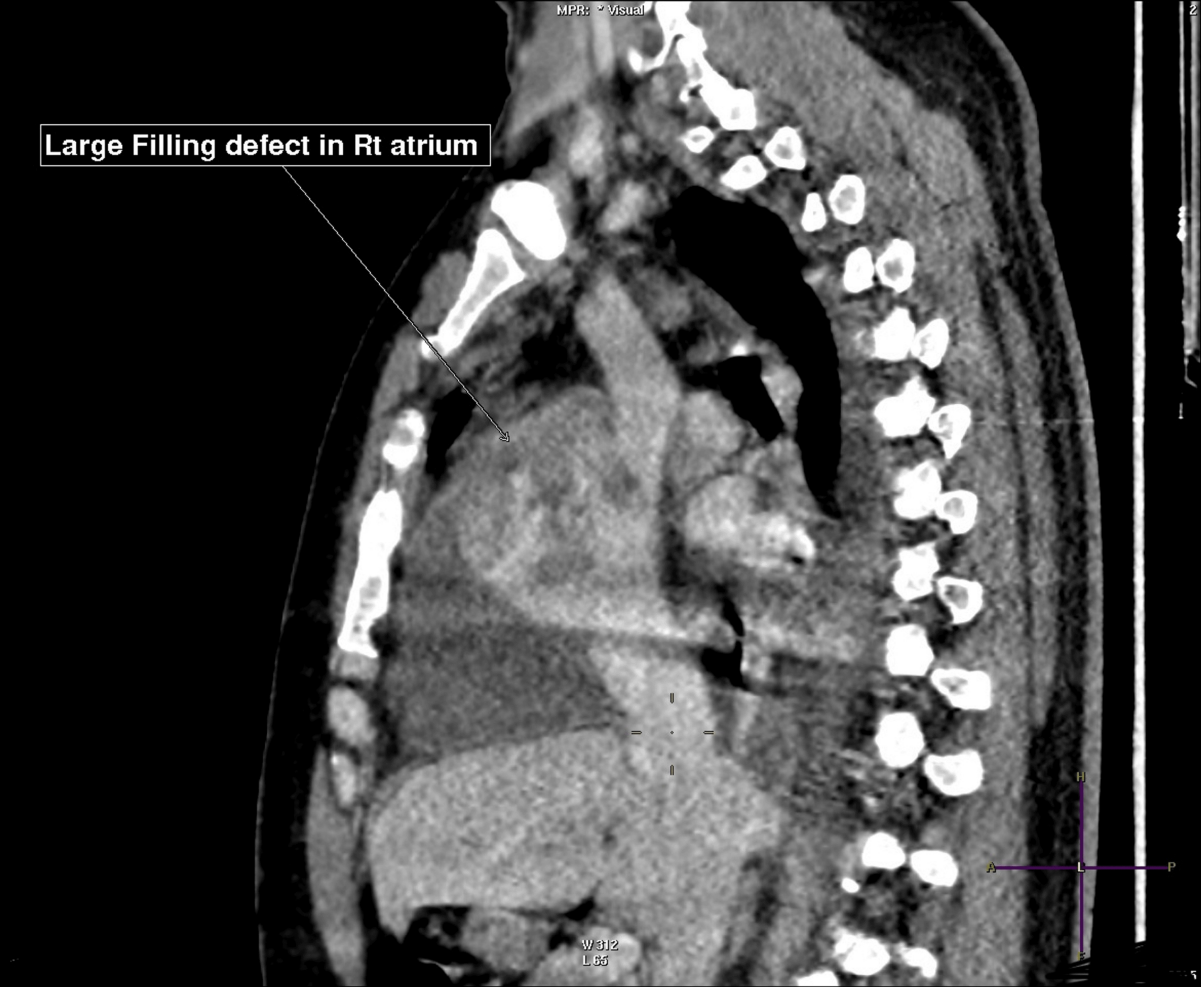
Computed tomography (CT) chest showing right atrial mass extending to the superior vena cava

CT pulmonary angiography confirmed multiple bilateral segmental pulmonary emboli, a right atrial filling defect extending toward the superior vena cava, bilateral pleural effusions, and atelectasis. Staging CT of the chest, abdomen, and pelvis demonstrated no distant metastases (Figures 4, 5) [9].

**Figure 6:**
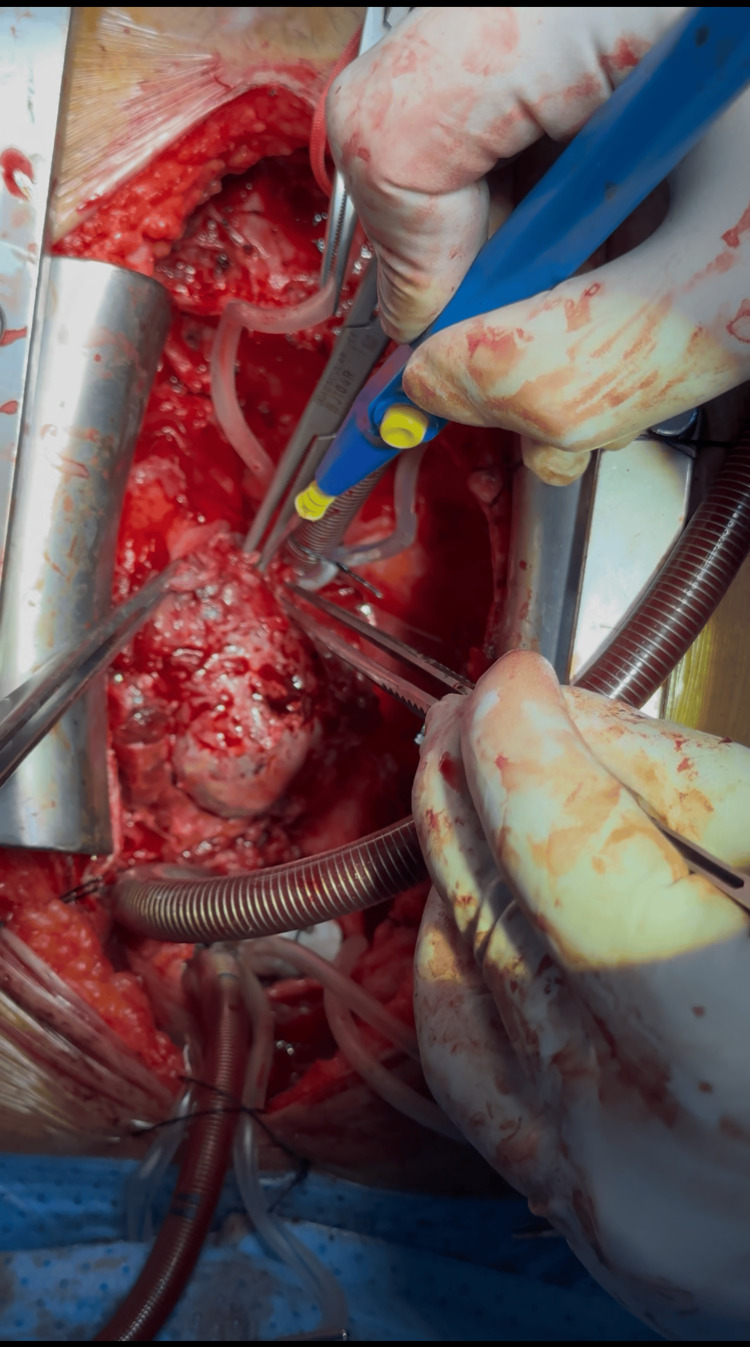
Intraoperative view showing the mass

Given the persistent mass and recurrent effusion, the patient underwent open-heart surgery on August 24, 2025, which included excision of the right atrial mass and part of the anterior wall, reconstruction with a bovine pericardial patch, creation of a pericardial window, mediastinal lymph node biopsy, and removal of organized right atrial thrombus (Figure 6).

**Figure 7:**
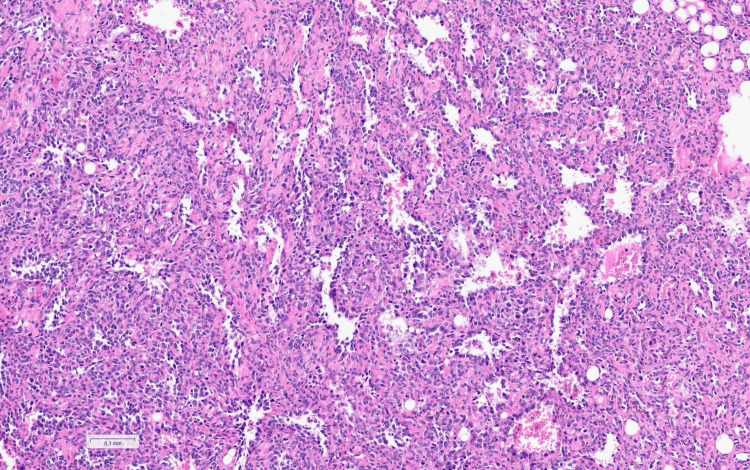
Hematoxylin and eosin stain shows numerous irregularly shaped anastomosing vascular channels lined by atypical endothelial cells

**Figure 8:**
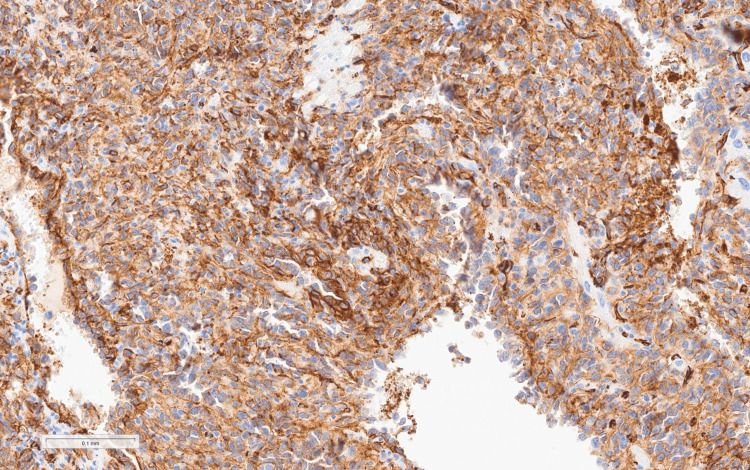
CD31 immunostain shows strong vascular marker expression

Histopathological examination demonstrated a malignant spindle cell neoplasm consistent with angiosarcoma involving the right atrium, pericardium, and thrombus, while mediastinal lymph nodes were reactive without metastasis (Figures 7, 8) [6,7].

Postoperatively, the patient remained hemodynamically stable. Chest drains were removed on day 10, and he was discharged on September 4, 2025, on apixaban 5 mg twice daily with a plan for systemic chemotherapy.

## Discussion

Right atrial angiosarcoma is a highly aggressive malignancy that often presents with non-specific symptoms, pericardial effusion, or cardiac tamponade due to tumor bleeding [5]. Delayed diagnosis remains common because early clinical manifestations are subtle and overlap with many benign cardiac conditions [6]. This patient’s unusual presentation with abdominal pain further highlights the diagnostic difficulty associated with this disease.

Key diagnostic elements

Hemorrhagic exudative tamponade is a characteristic finding in cardiac angiosarcoma and reflects active tumor hemorrhage [10]. A large right atrial mass with extension toward the superior vena cava is a classic imaging feature and is best demonstrated by CT and MR imaging [7,9]. Pulmonary embolism may occur as a result of tumor thrombus fragmentation or invasion into the venous circulation [6]. Histopathologic confirmation remains the diagnostic gold standard and shows malignant endothelial proliferation consistent with angiosarcoma [2,4].

Role of surgery and systemic therapy

Surgical excision plays a critical role by providing definitive tissue diagnosis, relieving life-threatening tamponade, and preventing further embolic complications [7,8]. However, recurrence is common due to the infiltrative nature of the tumor and early micrometastatic spread [6,8]. Therefore, systemic therapy remains essential, with both doxorubicin- and paclitaxel-based regimens demonstrating clinical activity in advanced disease [3].

Prognosis and comparison with previous cases

Primary cardiac angiosarcoma carries a poor prognosis, with median survival ranging from six to 12 months from diagnosis, largely due to delayed detection and early metastatic spread [5,6]. Comparison with previously reported cases shows that patients presenting with cardiac tamponade or pulmonary embolism, as in this report, represent a particularly high-risk subgroup requiring urgent intervention [7-9]. Our patient’s clinical course, including early surgical excision and planned systemic therapy, aligns with outcomes reported in similar cases, though long-term survival remains uncertain.

Limitations

This report is limited by the absence of long-term follow-up data beyond hospitalization and the lack of molecular or genomic profiling, which could provide insight into potential targeted therapeutic options. Additionally, being a single case, it cannot capture the full spectrum of disease variability or treatment response.

Future directions

Further research is needed to identify strategies for earlier detection, optimize surgical and systemic therapy combinations, and explore molecularly targeted treatments. Integration of genomic profiling and multi-institutional registries may help improve prognosis and guide personalized management for patients with this rare malignancy.

## Conclusions

Right atrial angiosarcoma should be considered in patients presenting with hemorrhagic pericardial effusion, intracardiac masses, or unexplained pulmonary emboli. Early multimodality imaging, pericardiocentesis, and timely surgical intervention are essential for diagnosis and hemodynamic stabilization. Multimodality oncologic treatment, including systemic therapy, remains critical given the tumor’s aggressive nature.

Although long-term follow-up and molecular profiling were not available in this case, reporting such rare presentations contributes valuable clinical and educational insight. Future studies incorporating genomic characterization and extended outcome monitoring may help guide personalized management and improve prognosis in this challenging disease.
